# Using Ultraviolet C (UVC) in Operating Rooms: A Hygiene Improvement

**DOI:** 10.1017/ash.2021.123

**Published:** 2021-07-29

**Authors:** Roberta Bosco, Gabriele Messina, Davide Amodeo, Gabriele Cevenini, Simona Gambelli

## Abstract

**Background:** Disinfection procedures are an essential aspect of preventing cross contamination, especially in situations where the risk of infection is higher, such as in operating rooms (ORs). Disinfection procedures in ORs at the end of each surgery session are not the same as final cleaning procedures. We assessed the difference in microbial contamination between different levels of disinfection, before T(0) and after T(1) the use of an ultraviolet C device (UVC-D). **Methods:** A cross-sectional study was conducted between December 2019 and August 2020 in a private clinic. Three sanitation levels (SL1–SL3) were compared for the reduction in colony-forming units (CFU) between T(0) and T(1): (1) no disinfection after surgery (SL1);, (2) after in-between cleaning (SL2), and (3) after terminal cleaning (SL3). UVC-D was used for 6 minutes, 3 minutes per bed side. Overall, 260 Petri dishes were used in 3 ORs, incubated at 36°C, and CFU were counted after 48 hours. Descriptive statistics, Wilcoxon test, and MANOVA for repeated measures were performed to verify the 95% statistical difference between T(0) and T(1), both on the whole sample and combined with the different SLs. **Results:** The unstratified analysis showed statistically significant differences (Wilcoxon test, p < 0.05) between T(0) and T(1), with means and standard deviations of 11.42 ± SD 41.19 CFU/PD and 5.91 ± SD 30.89, respectively. The Manova test for repeated measures, applied to 54 pairs of measurements, showed no significant difference between SLs in T(0)-T(1) CFU reduction. Overall, the mean percent reduction in CFU was 93.48% (CI95% = 86.97-99.99%). **Conclusions:** The results showed significant improvements in disinfection under any condition tested with UVC-D. Using the device immediately after surgery (SL1), before standard cleaning procedures, reduced CFUs by 97.3%. In some situations, UVC light was sufficient to reduce CFU to zero, even without chemical and mechanical cleaning. However, we do not recommend this approach; UVC light disinfection should be applied only after sanitization procedures because it does not remove dirt.

**Funding:** UltraViolet Device, Inc

**Disclosures:** None

